# Cancer-Related Distress: How Often Does It Co-occur With a Mental Disorder? – Results of a Secondary Analysis

**DOI:** 10.3389/fpsyg.2021.660588

**Published:** 2021-06-23

**Authors:** Jochen Ernst, Michael Friedrich, Sigrun Vehling, Uwe Koch, Anja Mehnert-Theuerkauf

**Affiliations:** ^1^Department of Medical Psychology and Medical Sociology, University Hospital Leipzig, Leipzig, Germany; ^2^Department of Medical Psychology, University Medical Center Hamburg-Eppendorf, Hamburg, Germany

**Keywords:** cancer, distress, distress thermometer, screening, mental disorders, CIDI, psycho-oncology, ROC

## Abstract

**Objectives:**

The Distress Thermometer (DT) is a validated and widely used screening tool to identify clinically relevant distress in cancer patients. It is unclear, to which extend subjectively perceived distress measured by the DT is related to objective burden (mental disorder). We therefore examine the co-occurrence of a mental disorder for different DT thresholds and explore the diagnostic properties of the DT in detecting a mental disorder.

**Methods:**

In this multicenter cross-sectional study, we included 4,020 patients with mixed cancer diagnoses. After selection of relevant cases, weighting procedure and imputation of missing data we evaluated the data of *N* = 3,212 patients. We used the DT to assess perceived distress and the standardized Composite International Diagnostic Interview for Oncology (CIDI-O) to assess the 4-week prevalence of mental disorders. The association between distress and any mental disorder (MD) is calculated using Pearson correlations. Relative risks for MD in patients with/without distress and the co-occurrence of distress and MD were calculated with Poisson regression. To assess the operating characteristics between distress and MD, we present the area under the curve (AUC).

**Results:**

22.9% of the participants had a cut-off DT level of ≥5 and were affected by MD. Each level of distress co-occurs with MD. The proportion of patients diagnosed with MD was not greater than the proportion of patients without MD until distress levels of DT = 6 were reached. The correlation between DT and MD was *r* = 0.27. The ROC-analysis shows the area under curve (AUC) = 0.67, which is classified as unsatisfactory. With increasing distress severity, patients are not more likely to have a mental disorder.

**Conclusion:**

Our results suggests viewing and treating cancer-related distress as a relatively distinct psychological entity. Cancer-related distress may be associated with an increased risk for a mental disorder and vice versa, but the overlap of both concepts is very moderate.

## Introduction

The Distress Thermometer (DT) is one of the most widely used tools to identify patients with clinically relevant distress in the cancer setting (Donovan et al., 2014). It is recommended as a screening instrument for distress by many clinical guidelines in cancer care (Carlson et al., 2012; Deutsche Krebsgesellschaft et al., 2014; Wuller et al., 2017; National Comprehensive Cancer Network, 2020). Its purpose as an ultra-short screening measure is closely linked to its practicality in routine oncology care (Pirl et al., 2014). Ownby (2019) shows in her current review, that the DT is a tool with well-established validity and brevity that is available in multiple languages.

Distress, as assessed by the DT, is conceptualized as multifactorial unpleasant experience (e.g., a cancer diagnosis) that may interfere with coping skills, ranging from common feelings of sadness and fears to severe reactions that can be diagnosed as psychiatric illnesses (Riba et al., 2019). Yet, the DT’s performance as a screening tool is sometimes evaluated in terms of detecting the occurrence of a mental disorder (Recklitis et al., 2016; Schaffeler et al., 2017). This conceptual vagueness is also reflected by the empirical questions regarding the extent to which emotional distress co-occurs with a diagnosis of a mental disorder (objective burden), or reflects psychological problems that are related to single symptoms of mental disorders.

Related to this is the question of the extent to which increasing severity of distress can also be expected to overlap with mental disorders.

Mitchell’s (2007) meta-analysis concluded that while the Distress Thermometer may not perform well in detecting cases of mood, anxiety, and adjustment disorders, it performs reasonably well in excluding cases of mental disorders. Further studies presented mixed results, with poor to good discrimination performance of the DT (AUC’s ranging from 0.66 to 0.89) (Grassi et al., 2009; Thekkumpurath et al., 2009; Patel et al., 2011; Wang et al., 2011; Ryan et al., 2012; Recklitis et al., 2016; Cruzado and Hernandez-Blazquez, 2018). Although results seem to point to a limited co-occurrence of distress and mental disorders, the pattern remains inconclusive.

### Objectives

We aimed to analyze the relationship of cancer-related distress (as assessed by the DT) with the 4-week-prevalence of any mental disorder (MD) assessed the standardized computer-assisted Composite International Diagnostic Interview for mental disorders adapted for cancer patients (CIDI-O). The article deals with the following study aims:

1. to estimate the association (correlation) between distress and MD (objective burden),
2. to examine the co-occurrence of MD and distress for different DT thresholds,
3. to explore the diagnostic properties of the DT in detecting any MD.

## Materials and Methods

### Participants and Procedures

We use data from a large epidemiological cross-sectional study (Mehnert et al., 2012, 2014). Patients were recruited while receiving treatment from oncological inpatient clinics at acute care hospitals, specialized outpatient cancer care facilities, and cancer rehabilitation centers across Germany. Exclusion criteria were age younger than 18 or older than 75 years, severe cognitive or physical impairment, and language barrier. After providing written informed consent, participants were screened with the Patient-Health-Questionnaire-9 (PHQ-9). Patients with sum scores ≥9 were further assessed by a standardized diagnostic interview for mental disorders (CIDI-O). Patients with sum scores <9 were randomly assigned to the interview. All patients completed a set of self-report questionnaires. Further details are published in the study protocol (Mehnert et al., 2012). The research ethics committees of the local medical association in each study center approved this study.

**FIGURE 1:**
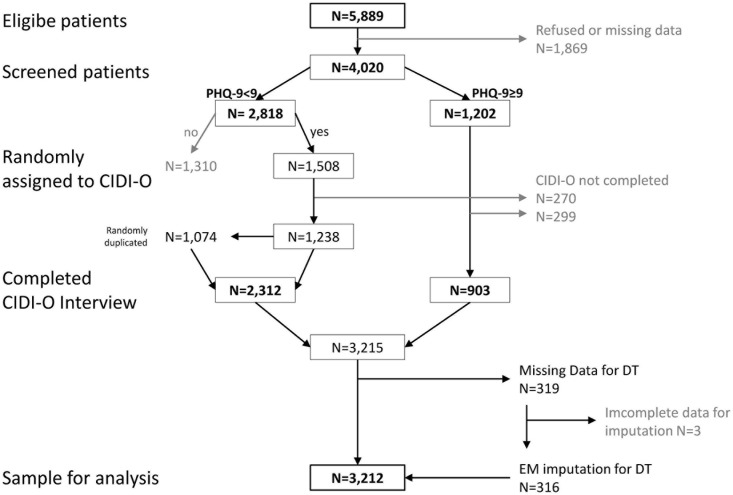
Flowchart of the sample (patient recruitment, weighting, imputation).

Of 5,889 eligible patients, 4,020 (68%) agreed to participate in the study (Figure 1). Reasons for non-participation were especially lack of interest (55%) or symptom burden (33%) (a more detailed description of the sample is given by Hartung et al. (2017). As reported elsewhere (Mehnert et al., 2014), non-participants were younger, but did not differ in gender. 1,202 patients screened ≥9 on the PHQ-9 and were assigned to the *Composite International Diagnostic Interview for Oncology* (CIDI-O), of which 903 completed the interview. 2,818 patients screened <9 on the PHQ-9, of which 1,508 were randomly assigned to the CIDI-O because of economic reasons. Among these, 1,238 completed the interview. The group with PHQ-9 < 9 was underrepresented in the raw data set due to the random assignment to the interview. To correct this, we weighted those by randomly duplicating 1,074 cases from the 1,508 participants who completed the CIDI-O interview based on the exclusion/inclusion ratio at randomization (1,310 excluded/1,508 randomly assigned to CIDI-O). This lead to a sample of *N* = 3,215 cases.

### Measures

Demographic data were collected by a standardized questionnaire. Disease-related characteristics were obtained from medical charts.

We used the standardized computer-assisted *Composite International Diagnostic Interview for Oncology* (CIDI-O) to assess the 4-week prevalence of mental disorders resulting from general medical condition, substance use disorders, mood disorders, anxiety disorders, somatoform disorders, and eating disorders (Hund et al., 2014). The CIDI-O further enables diagnosis of adjustment disorders in response to specific cancer-related stressors (Hund et al., 2016). In accordance with DSM-IV criteria, adjustment disorder was diagnosed where distress was problematic, out of proportion to the clinical setting and causing impairment, no other axis I disorder was present, and symptoms did not persist for longer than 6 months. The following analyses were based on the binary coded variable *any mental disorder – MD* (at least one mental disorder mentioned above is present vs. none). The largest proportions in MD in our study have the following disorders: any anxiety disorder (11.5%), adjustment disorder (11.1%) and any mood disorder (6.5%) [detailed information are delivered in Mehnert et al. (2014)].

With the Distress Thermometer (DT) we assessed the global level of distress experienced in the past week on an 11-point visual analog scale ranging from 0 (“no distress”) to 10 (“extreme distress”). This instrument is validated for the use in the German language with the recommended cut-off ≥5 to detect cancer-related distress (Mehnert et al., 2006).

### Handling of Missing Data

Missing data in the DT were estimated with the Expectation Maximization (EM) algorithm (Dempster et al., 1977) using IBM SPSS Statistics. 319 patients (9.9%) did not answer the DT. To estimate these missing values we included age, gender, MD (no missing values), and additionally the items of the PHQ-9, as well as the items of the Hospital Anxiety and Depression Scale (HADS). The HADS consists of seven items measuring depression and seven items measuring anxiety. All items are rated on a 5-point Likert scale. Both questionnaires are widely used as screening instruments in patients with cancer (Mitchell et al., 2010; Thekkumpurath et al., 2011). Subjects who did not answer at least 50% of the items of a scale (PHQ-9, HADS-depression, or HADS-anxiety) were excluded (*N* = 3). Imputed values out of the possible item-range were set to the nearest possible value.

Thus the *resulting sample size* was *N* = 3,212. Figure 1 presents a flowchart from the sample of eligible patients to the sample for analysis.

### Data Analysis

The prevalence rates for mental disorders and distress were calculated descriptive, accompanied by the 95% confidence intervals (95% CI) based on 1,000 bootstrap samples. Prevalence rates for MD are displayed for different levels of distress severity in tabular and visual form. The association between MD and distress [cut-off ≥ 5, (Mehnert et al., 2006)] is calculated using Pearson correlations.

To classify correlation based effect sizes we used the recommendations from Cohen (1988) (*r* = 0.1 small effect, *r* = 0.3 medium, *r* = 0.5 large).

We calculated the relative risks (RR) for MD in patients with distress relative to those without distress to estimate the co-occurrence of distress and MD. For this we used Poisson regression with robust standard errors. Analyses were repeated for different DT cut-off scores to determine whether higher distress thresholds were associated with a higher risk for MD.

To illustrate the operating characteristics between distress (test) and MD (standard), we present the area under the curve (AUC) which corresponds to the probability for the correct identification of the standard by the test (Hanley and McNeil, 1982).

Furthermore we present the ratio of true positives to all positive conditions (sensitivity, SEN), the ratio of true negatives to all negative conditions (specificity, SPE), the diagnostic ability or maximum difference between sensitivity and false positive rate (Youden Index), the ratio of true positives to all positive predictions (positive predictive value, PPV), as well as the negative predictive value (NPV). To classify the accuracy by the AUC we used the cut-offs presented by Zhu et al. (2010) (AUC ≥ 0.6 not good, AUC ≥ 0.7 worthless, AUC ≥ 0.8 good, AUC ≥ 0.9 excellent).

### Software

Statistical analyses were conducted with IBM SPSS Statistics 26, R (R Development Core Team, 2015) and Microsoft Excel 2010.

## Results

### Sample Characteristics and Descriptive Analysis of Distress and Mental Disorders

As shown in Table 1, 1,103 (51.5%) of 2,141 patients were female. On average participants were 57.6 years old (range 18–75 years), and the most frequent tumor entities were breast (20.6%), prostate (14.9%), and digestive organs (21.3%).

**TABLE 1:** Sample characteristics.

**Variable**	***N* (%)**
Age, mean (SD, Range)	57.6 (11.1, 18–75)
**Gender**	
Female	1,103 (51.5)
Male	1,038 (48.5)
Marital status – married	1,358 (63.4)
**Education**	
Less than high school	1,475 (68.9)
High school (12–13 years)/University	666 (31.1)
**Tumor diagnosis**	
Digestive organs	456 (21.3)
Breast	442 (20.6)
Prostate	318 (14.9)
Respiratory and intrathoracic organs	201 (9.4)
Female genital organs	183 (8.5)
Diseases of the blood and blood-forming organs	170 (7.9)
Urinary tract	128 (6.0)
Lip, oral cavity and pharynx	62 (2.9)
Eye, brain and other parts of central nervous system	41 (1.9)
Skin	39 (1.8)
Mesothelial and soft tissue	38 (1.8)
Other	63 (2.9)
Time since diagnosis (month), mean (SD, range)	13.5 (24.9, 0–318)
up to 3 month	747 (41.2)
**ECOG Performance Status (ECOG/WHO Score)**	
0 (Fully active)	986 (47.4)
1 (Restricted in physically strenuous activity)	757 (36.4)
2 (Up and about more than 50% of waking hours)	260 (12.5)
3 (Confined to bed or chair more than 50% of waking hours)	76 (3.7)
4 (Completely disabled)	3 (0.1)
**Treatment setting**	
Inpatient	932 (43.5)
Outpatient	640 (29.9)
Rehabilitation, inpatient	569 (26.6)

*Unweighted sample, *N* = 2,141.

### Association Between Distress and MD

**FIGURE 2:**
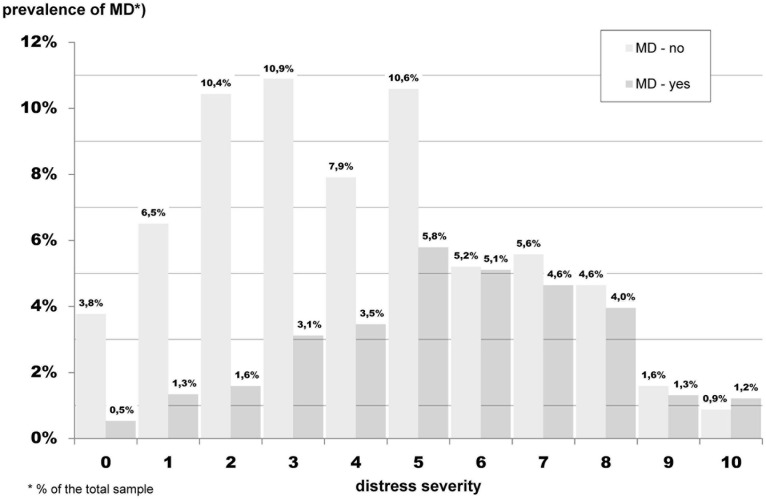
Frequency of distress for patients with (dark gray) and without MD (light gray) for different levels of distress severity (weighed sample, *N* = 3,212).

Figure 2 presents the ratio of patients diagnosed with MD (dark gray) to those without MD (light gray). Shown are the total percentages for MD. For example, 5.8% of the total sample has a distress level = 5 and a MD, and 10.6% of the total sample has distress level = 5 and no MD.

As shown, 22.9% of the participants had a cut-off DT level of ≥5 and were affected by MD, and 10.0% had a DT level of <5 and were affected by MD. It is seen, that from DT level ≥6, the proportion of both groups (with and without MD) is similar, that is the ratio of patients with MD to patients without MD ranges near one. The overlap of distress and MD is rather small. The correlation of both concepts shows an *r* = 0.27 (95% CI 0.23–0.31, *p* < 0.001). For more information regarding correlation of distress and mental disorder (based on mental disorder groups; see Supplementary Table 1).

### Co-occurrence of Distress With Mental Disorders (MD) for Different Distress Thresholds

Table 2 shows cross-tabulations for the number of patients with distress vs. the number of patients with/without MD for each distress level. For instance, distress threshold ≥5 means that 1,645 (51%) patients are considered as not distressed, and 1,567 (49%) patients are considered as distressed. From these, 698 (22%) patients were diagnosed with MD. In addition, the relative risk for MD associated with increasing cut-offs of distress is presented. For example, distress ≥5 was associated with a 2-fold increased risk for diagnosis of MD (RR = 2.1, 95% CI 1.8–2.5). Approximately 32% (*N* = 1,029) of patients were diagnosed with at least one mental disorder (MD) and *N* = 1,051 (49%) were distressed vs. *N* = 1,645 (51%) were not distressed.

**TABLE 2:** Co-occurrence of distress cases with MD cases for increasing distress thresholds.

**Distress threshold**	**Distress**	**Any Mental Disorder (MD)** Total = 3212*		**Relative Risk for Any Mental Disorder Associated with Distress (95% CI)**
		**No 2,183 (68%)**	**Yes 1,029 (32%)**	
**≥1**	Not Distressed: 145 (5%)	126 (4%)	19 (1%)	2.5 (1.6–3.8)
	Distressed: 3,067 (95%)	2,057 (64%)	1,010 (31%)	
**≥2**	Not Distressed: 416 (13%)	353 (11%)	63 (2%)	2.3 (1.8–2.9)
	Distressed: 2,796 (87%)	1,830 (57%)	966 (30%)	
**≥3**	Not Distressed: 835 (26%)	716 (22%)	119 (4%)	2.7 (2.3–3.2)
	Distressed: 2,377 (74%)	1,467 (46%)	910 (28%)	
**≥4**	Not Distressed: 1,269 (40%)	1,040 (32%)	229 (7%)	2.3 (2.0–2.6)
	Distressed: 1,943 (60%)	1,143 (36%)	800 (25%)	
**≥5**	Not Distressed: 1,645 (51%)	1,314 (41%)	331 (10%)	2.2 (2.0–2.5)
	Distressed: 1,567 (49%)	869 (27%)	698 (22%)	
**≥6**	Not Distressed: 2,161 (67%)	1,634 (51%)	527 (16%)	2.0 (1.8–2.2)
	Distressed: 1,051 (33%)	549 (17%)	502 (16%)	
**≥7**	Not Distressed: 2,485 (77%)	1,797 (56%)	688 (21%)	1.7 (1.5–1.9)
	Distressed: 727 (23%)	386 (12%)	341 (11%)	
**≥8**	Not Distressed: 27,99 (87%)	1,966 (61%)	833 (26%)	1.6 (1.4–1.8)
	Distressed: 413 (13%)	217 (7%)	196 (6%)	
**≥9**	Not Distressed: 3,064 (95%)	2,111 (66%)	953 (30%)	1.7 (1.4–1.9)
	Distressed: 148 (5%)	72 (2%)	76 (2%)	
**≥10**	Not Distressed: 3,152 (98%)	2,159 (67%)	993 (31%)	1.9 (1.5–2.4)
	Distressed: 60 (2%)	24 (1%)	36 (1%)	

*% of the total sample; weighed sample.

### Diagnostic Properties of the DT in Detecting Any Mental Disorder (MD)

**FIGURE 3:**
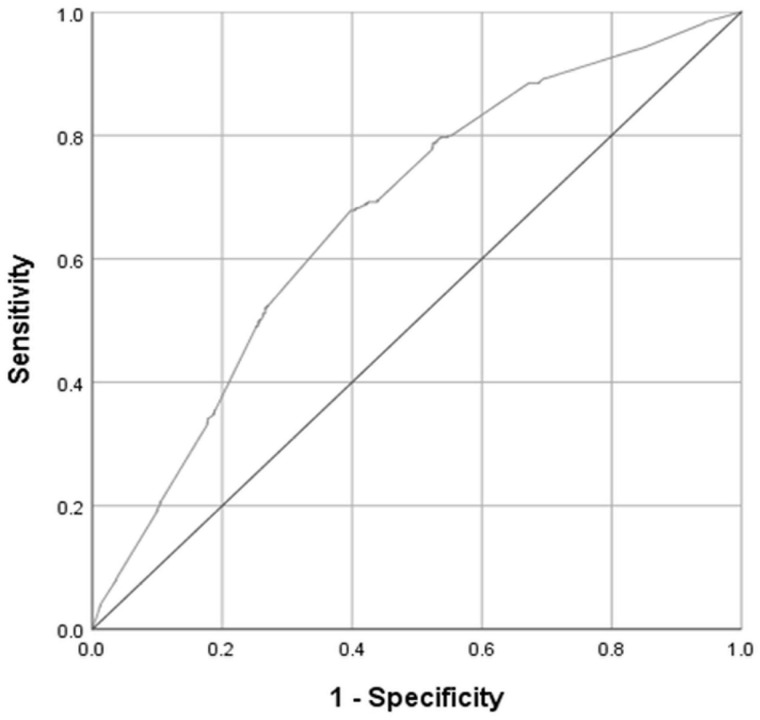
Receiver operating characteristic (ROC) curve for the 1-item Distress Thermometer (DT), reference standard: any mental disorder (MD). Area under the ROC curve with 95% confidence interval (CI) is 0.67 (95% CI, 0.65–0.69; weighed sample, *N* = 3,212).

Table 3 presents the diagnostic properties of the DT for detection of MD for cut-offs with minimum of both SEN, respective SPE > 30. The prevalence of MD was 1,029 (32.0), 95% CI 30.4–33.7, and the area under curve (AUC) was 0.67, 95% CI 0.65–0.69 (Figure 3).

**TABLE 3:** Diagnostic properties of the DT for detection of MD *, **.

**Cut off (distress≥)**	**Value (95% CI)**				
	**SEN**	**SPE**	**Youden J**	**PPV**	**NPV**
3	88 (86–90)	33 (31–35)	0.21 (0.20–0.23)	38 (36–40)	86 (83–88)
4	78 (75–80)	48 (46–50)	0.26 (0.24–0.27)	41 (39–43)	82 (80–84)
5	68 (65–71)	60 (58–62)	0.28 (0.26–0.30)	44 (42–47)	80 (78–82)
6	49 (46–52)	75 (73–77)	0.24 (0.22–0.25)	48 (45–51)	76 (74–77)
7	33 (30–36)	82 (81–84)	0.15 (0.14–0.17)	47 (43–51)	72 (71–74)

*Weighted sample, *N* = 3,212.

**SEN, sensitivity; SPE, specificity; Youden J, Youden Index; PPV, positive predictive value; NPV, negative predictive value; CI, confidence interval.

Choosing the cut-off DT ≥ 5, the Table 3 shows that 68% from the patients with MD were identified as positive (sensitivity), and 60% from the patients who did not have MD were correctly identified as negative (specificity). The Youden Index has its maximum for this cut-off (J = 0.28) and indicates the best choice of equally important sensitivity and specificity. For patients who reach the cut-off the probability for truly having the positive condition is 0.44. Among those patients with a DT level ≥5, 44% have a MD (positive predictive value). For patients who were screened below this cut-off, the probability for not having any mental disorder was 80% (negative predictive value).

## Discussion

The aim of the study was to investigate the relationship between subjectively perceived stress (measured with the DT) and the occurrence of any mental disorder (MD), measured with the CIDI-O). For this purpose, we examined the co-occurrence of MD for different DT thresholds and explore the diagnostic properties of the DT in detecting MD. We evaluated the data of *N* = 3,212 cancer patients (after weighting and imputation procedures).

The prevalence of MD was estimated with 32%, the prevalence of distress (DT level ≥5) with nearly 49%. 22.9% of the participants with DT level ≥ 5 were considered as accompanied by MD and distress. The association of distress and mental disorder is small (*r* < 0.3).

We found, that each level of distress co-occurs with a diagnosis of MD. Although the proportion of patients diagnosed with MD was not greater than the proportion of patients without a mental disorder until distress levels of DT = 6 were reached. Visually, one could see that from a DT level ≥6, the proportion of both groups is similar, that is the ratio of patients with MD to patients without MD ranges near one. Furthermore the maximum prevalence for MD is already reached for patients with a distress level of DT = 5.

Altogether, patients who reached the threshold that corresponds to the respective distress level showed approximately a two times higher risk of being diagnosed with MD than patients who scored below this threshold. Hence, each level of distress reflects symptoms of mental disorders, but only for levels of DT ≥ 6 and following, the probability for showing these symptoms is nearly as probable as not showing them. Below these levels it is rather clearly less probable to show such symptoms.

From this perspective, the DT might be considered as a very rough screening tool for MD. In addition, the ROC-Analysis could – in line with other studies – not provide the best support for this application (Lycke et al., 2017; Cruzado and Hernandez-Blazquez, 2018). On the one hand, this is shown by the area under curve, which can be classified as “not good”, but it is shown also by the diagnostic properties. For example, with a cut-off of DT ≥ 3 we could identify 88% of the patients who have MD, but simultaneously we would wrongly identify 77% (=100-SPE) of the patients without MD as positive. In the end, the sample after screening would contain less than a half (PPV = 44%) of patients with MD. Even if we would choose a more strict cut-off like DT ≥ 7, this would not change (PPV = 47%). With increasing distress severity, patients are not more likely to have a mental disorder. That is, at the severe end of the distress continuum, diagnosis of a mental disorder (including adjustment disorder) is not more likely than at the lower end. This suggests that clinically relevant cancer-related distress may not be viewed as a downward extension of the “mental-disorder-category” by a “subthreshold-symptoms-category”, where the latter is distinguished from the former by lower symptom intensity and/or symptom count.

Yet, nearly a third of our sample reported clinically relevant distress in absence of any mental disorder. Those patients’ high distress scores may still reflect a genuine psychological burden, albeit not necessarily a maladaptive one, and indicate a need for support (Dekker et al., 2017). This suggests viewing and treating cancer-related distress more consequently as a distinct psychological entity. This also echoes the low co-occurrence of fear of cancer recurrence and anxiety disorders (Dinkel et al., 2014). This does not mean that cancer-related distress may not be associated with an increased risk for a mental disorder and vice versa. Rather, our results suggest that the divergence between these concepts may be greater than previously thought.

Practically, the supportive care needs associated with high levels of cancer-related distress may thus tend to diverge from those associated with a mental disorder (MD). This may not be surprising from a clinical point of view. However, considering the result that disorders seem to play a less prominent role in psychological consequences of cancer, while high distress is present in every second individual, framing interventions in the cancer setting with reference to psychiatric categories may not match entirely with patients’ needs. This may especially apply to the evaluation of psychosocial interventions, where a focus on the reduction of depression and anxiety symptoms may distract from some of their central effects.

It is important to note that these findings do not aim to draw conclusions about the effectiveness or usefulness of distress screening programs. Rather, they point toward the type of knowledge we may infer from a positive or negative screening result. This information may not primarily lie in detecting or ruling out a mental disorder or subthreshold distress. It may rather be more complex than previously thought. Development of cancer-specific psychosocial interventions may thus require considerable efforts to conceptualize relevant distress phenomena independently of psychiatric nosology (Dekker et al., 2017; Lebel et al., 2017).

### Limitations

Results describe average associations in a mixed population representative for cancer sites and treatment settings in Germany. Naturally, the association between distress and mental disorders will be somewhat closer in subgroups with a higher prevalence of disorders, such as women with breast cancer (42% prevalence of any disorder in this sample compared to average across tumor entities of 32%). A diagnosis-specific evaluation of the relationship between DT and MD would further differentiate our results.

### Practical Implications

In clinical practice, it is important to identify acute distress and psycho-oncological care needs in cancer patients as early as possible (e.g., using the DT). In addition, the medical history can provide clues to a pre-existing psychological disorder, which may be further exacerbated in the context of cancer diagnosis and treatment and therefore needs to be considered in the psycho-oncological care process.

In addition to ultra-short screening (e.g., DT), the use of a standard screening instrument may be considered when manifest psychological distress is suspected. Good evidence is available internationally for the Hospital Anxiety and Depression Scale (HADS). If a mental disorder is suspected, the patient can be diagnosed using a specific clinical diagnostic tool (e.g., the SCID – Structured Clinical Interview for DSM).

If possible, practitioners should always include well-known risk factors for an increased psychological stress reaction in the psycho-oncological diagnosis (“yellow flags”, e.g., younger female patients with younger children, low social status, comorbid psychological disorders, side effects of the therapy, chronic pain, etc.).

## Data Availability Statement

The raw data supporting the conclusions of this article will be made available by the authors, without undue reservation.

## Ethics Statement

The studies involving human participants were reviewed and approved by the study was approved by the following Medical Associations: Hamburg: Ref. Nr. 2768, Schleswig-Holstein: Ref.-Nr. 61/09; Freiburg: Ref.-Nr. 244/07, Heidelberg: Ref.-Nr. S-228/2007; 50155039; Würzburg: Ref. Nr. 107/07; Leipzig: Ref. Nr. 200–2007. The patients/participants provided their written informed consent to participate in this study.

## Author Contributions

AM-T and UK: conception and design. AM-T and MF: administrative support. JE, MF, AM-T, and SV: data analysis and interpretation. All authors: manuscript writing and final approval of manuscript.

## Conflict of Interest

The authors declare that the research was conducted in the absence of any commercial or financial relationships that could be construed as a potential conflict of interest.

## References

[B1] Carlson L. E. Waller A. Mitchell A. J. (2012). Screening for distress and unmet needs in patients with cancer: review and recommendations. *J. Clin. Oncol.* 30 1160–1177. 10.1200/jco.2011.39.5509 22412146

[B2] Cohen J. (1988). *Statistical Power Analysis for the Behavioral Sciences.* Hillsdale, NJ: Taylor and Francis.

[B3] Cruzado J. A. Hernandez-Blazquez M. (2018). Mental disorder screening on cancer patients before and after radiotherapy and at the 1-month follow-up. *Support Care Cancer* 26 813–821. 10.1007/s00520-017-3894-2 28942566

[B4] Dekker J. Braamse A. Schuurhuizen C. Beekman A. T. F. Van Linde M. Sprangers M. A. G. (2017). Distress in patients with cancer - on the need to distinguish between adaptive and maladaptive emotional responses. *Acta Oncol.* 56 1026–1029. 10.1080/0284186x.2017.1280848 28145789

[B5] Dempster A. P. Laird N. M. Rubin D. B. (1977). Maximum likelihood from incomplete data via the EM algorithm. *J. R. Stat. Soc. Series B Stat. Methodol.* 39 1–22.

[B6] Deutsche Krebsgesellschaft Deutsche Krebshilfe A.M.W.F (2014). *Psychoonkologische Diagnostik, Beratung und Behandlung von Erwachsenen Krebspatienten, Langversion 1.1 [Online].* Available online at: http://leitlinienprogramm-onkologie.de/Leitlinien.7.0.html (accessed January 10, 2021).

[B7] Dinkel A. Kremsreiter K. Marten-Mittag B. Lahmann C. (2014). Comorbidity of fear of progression and anxiety disorders in cancer patients. *Gen. Hosp. Psychiatry* 36 613–619. 10.1016/j.genhosppsych.2014.08.006 25213227

[B8] Donovan K. A. Grassi L. Mcginty H. L. Jacobsen P. B. (2014). Validation of the distress thermometer worldwide: state of the science. *Psycho Oncol.* 23 241–250. 10.1002/pon.3430 25160838

[B9] Grassi L. Sabato S. Rossi E. Marmai L. Biancosino B. (2009). Affective syndromes and their screening in cancer patients with early and stable disease: Italian ICD-10 data and performance of the Distress Thermometer from the Southern European Psycho-Oncology Study (SEPOS). *J. Affect. Disord.* 114 193–199. 10.1016/j.jad.2008.07.016 18757101

[B10] Hanley J. A. McNeil B. J. (1982). The meaning and use of the area under a receiver operating characteristic (ROC) curve. *Radiology* 143 29–36. 10.1148/radiology.143.1.7063747 7063747

[B11] Hartung T. J. Friedrich M. Johansen C. Wittchen H. U. Faller H. Koch U. (2017). The hospital anxiety and depression scale (HADS) and the 9-item patient health questionnaire (PHQ-9) as screening instruments for depression in patients with cancer. *Cancer* 123 4236–4243. 10.1002/cncr.30846 28654189

[B12] Hund B. Reuter K. Härter M. Brähler E. Faller H. Keller M. (2016). Stressors, symptom profile, and predictors of adjustment disorder in cancer patients. results from an epidemiological study with the composite international diagnostic interview, adaptation for oncology (Cidi-O). *Depress Anxiety* 33 153–161. 10.1002/da.22441 26474266

[B13] Hund B. Reuter K. Jacobi F. Siegert J. Wittchen H. U. Härter M. (2014). [Adaptation of the composite international diagnostic interview (CIDI) for the assessment of comorbid mental disorders in oncology patients: the CIDI-O]. *Psychother. Psychosom. Med. Psychol.* 64 101–107. 10.1055/s-0033-1357174 24343310

[B14] Lebel S. Ozakinci G. Humphris G. Thewes B. Prins J. Dinkel A. (2017). Current state and future prospects of research on fear of cancer recurrence. *Psychooncology* 26 424–427. 10.1002/pon.4103 26891602

[B15] Lycke M. Lefebvre T. Pottel L. Pottel H. Ketelaars L. Stellamans K. (2017). The distress thermometer predicts subjective, but not objective, cognitive complaints six months after treatment initiation in cancer patients. *J. Psychosoc. Oncol.* 35 741–757. 10.1080/07347332.2017.1365798 28816636

[B16] Mehnert A. Brähler E. Faller H. Härter M. Keller M. Schulz H. (2014). Four-week prevalence of mental disorders in patients with cancer across major tumor entities. *J. Clin. Oncol.* 32 3540–3546. 10.1200/jco.2014.56.0086 25287821

[B17] Mehnert A. Koch U. Schulz H. Wegscheider K. Weis J. Faller H. (2012). Prevalence of mental disorders, psychosocial distress and need for psychosocial support in cancer patients - study protocol of an epidemiological multi-center study. *BMC Psychiatry* 12:70. 10.1186/1471-244X-12-70 22747671 PMC3434016

[B18] Mehnert A. Müller D. Lehmann C. Koch U. (2006). Die deutsche Version des NCCN Distress-Thermometers. *Z. Psychiatr. Psychol. Psychother.* 54 213–223. 10.1024/1661-4747.54.3.213

[B19] Mitchell A. J. (2007). Pooled results from 38 analyses of the accuracy of distress thermometer and other ultra-short methods of detecting cancer-related mood disorders. *J. Clin. Oncol.* 25 4670–4681. 10.1200/JCO.2006.10.0438 17846453

[B20] Mitchell A. J. Meader N. Symonds P. (2010). Diagnostic validity of the Hospital Anxiety and Depression Scale (HADS) in cancer and palliative settings: a meta-analysis. *J. Affect. Disord.* 126 335–348. 10.1016/j.jad.2010.01.067 20207007

[B21] National Comprehensive Cancer Network (2020). *NCCN Clinical Practice Guidelines in Oncology: Distress Management. Version 3.2013 [Online].* Fort Washington, PA: National Comprehensive Cancer Network. Available online at: https://www.nccn.org/ (accessed January 07, 2021). 10.6004/jnccn.2019.0048 PMC6907687 31590149

[B22] Ownby K. K. (2019). Use of the distress thermometer in clinical practice. *J. Adv. Pract. Oncol.* 10 175–179. 31538028 PMC6750919

[B23] Patel D. Sharpe L. Thewes B. Bell M. L. Clarke S. (2011). Using the distress thermometer and hospital anxiety and depression scale to screen for psychosocial morbidity in patients diagnosed with colorectal cancer. *J. Affect. Disord.* 131 412–416. 10.1016/j.jad.2010.11.014 21130501

[B24] Pirl W. F. Fann J. R. Greer J. A. Braun I. Deshields T. Fulcher C. (2014). Recommendations for the implementation of distress screening programs in cancer centers: report from the American Psychosocial Oncology Society (APOS), Association of Oncology Social Work (AOSW), and Oncology Nursing Society (ONS) joint task force. *Cancer* 120 2946–2954. 10.1002/cncr.28750 24798107

[B25] R Development Core Team (2015). *R: A Language and Environment for Statistical Computing.* Vienna: R Development Core Team.

[B26] Recklitis C. J. Blackmon J. E. Chang G. (2016). Screening young adult cancer survivors for distress with the Distress Thermometer: comparisons with a structured clinical diagnostic interview. *Cancer* 122 296–303. 10.1002/cncr.29736 26457669 PMC4707976

[B27] Riba M. B. Donovan K. A. Andersen B. Braun I. Breitbart W. S. Brewer B. W. (2019). Distress management, version 3.2019, NCCN clinical practice guidelines in oncology. *J. Natl. Compr. Cancer Netw.* 17 1229–1249. 10.6004/jnccn.2019.0048 PMC6907687 31590149

[B28] Ryan D. A. Gallagher P. Wright S. Cassidy E. M. (2012). Sensitivity and specificity of the Distress Thermometer and a two-item depression screen (Patient Health Questionnaire-2) with a ‘help’ question for psychological distress and psychiatric morbidity in patients with advanced cancer. *Psycho Oncol.* 21 1275–1284. 10.1002/pon.2042 21919118

[B29] Schaffeler N. Sedelmaier J. Mohrer H. Ziser K. Ringwald J. Wickert M. (2017). [Patient’s autonomy and information in psycho-oncology: computer based distress screening for an interactive treatment planning (ePOS-react)]. *Psychother. Psychosom. Med. Psychol.* 67 296–303. 28719921 10.1055/s-0043-113438

[B30] Thekkumpurath P. Venkateswaran C. Kumar M. Newsham A. Bennett M. I. (2009). Screening for psychological distress in palliative care: performance of touch screen questionnaires compared with semistructured psychiatric interview. *J. Pain Symptom Manag.* 38 597–605. 10.1016/j.jpainsymman.2009.01.004 19692204

[B31] Thekkumpurath P. Walker J. Butcher I. Hodges L. Kleiboer A. O’connor M. (2011). Screening for major depression in cancer outpatients: the diagnostic accuracy of the 9-item patient health questionnaire. *Cancer* 117 218–227. 10.1002/cncr.25514 20737537

[B32] Wang G.-L. Hsu S.-H. Feng A.-C. Chiu C.-Y. Shen J.-F. Lin Y.-J. (2011). The HADS and the DT for screening psychosocial distress of cancer patients in Taiwan. *Psycho Oncol.* 20 639–646. 10.1002/pon.1952 21626611

[B33] Wuller J. Kuttner S. Foldenauer A. C. Rolke R. Pastrana T. (2017). Accuracy of the Distress Thermometer for home care patients with palliative care needs in Germany. *Palliat. Support Care* 15 288–294. 10.1017/s1478951516000699 27666282

[B34] Zhu W. Zeng N. Wang N. (2010). *Sensitivity, Specificity, Accuracy, Associated Confidence Interval and ROC Analysis with Practical SAS^®^ Implementations.* Baltimore, MD: NorthEast SAS users group, health care and life sciences.

