# Supplementary material for: Cancer-Related Distress: How Often Does It Co-occur With a Mental Disorder? – Results of a Secondary Analysis
Source: Front Psychol. 2021 Jun 23;12:660588. doi: 10.3389/fpsyg.2021.660588 (PMC8260981; doi:10.3389/fpsyg.2021.660588)
Supplement: Supplementary file 1 [file Table_1.docx]

**Table S1:** Correlations (Pearson’s r) of distress with mental disorders, weighted sample (N = 3,212)

| **Mental disorder** | **r ^*)^** |
| --- | --- |
| any anxiety disorder | 0.17 |
| any mood disorder | 0.20 |
| any adjustment disorder | 0.10 |
| any somatoform disorder | 0.10 |
| any mental disorder resulting from general medical condition | 0.10 |

^*)^ All correlations are significant with type-I-error probability p < 0.001.
